# Low-Temperature Atomic Layer Deposited Oxide on Titanium Nitride Electrodes Enables Culture and Physiological Recording of Electrogenic Cells

**DOI:** 10.3389/fnins.2020.552876

**Published:** 2020-09-18

**Authors:** Michele Dollt, Miriam Reh, Michael Metzger, Gerhard Heusel, Martin Kriebel, Volker Bucher, Günther Zeck

**Affiliations:** ^1^Natural and Medical Sciences Institute at the University of Tübingen, Reutlingen, Germany; ^2^Graduate School of Neural Information Processing/International Max Planck Research School, University of Tübingen, Reutlingen, Germany; ^3^Mechanical and Medical Engineering, Hochschule Furtwangen University, Villingen-Schwenningen, Germany

**Keywords:** atomic-layer deposition, microelectrode array, neuron, cardiomyocyte, action potential, CMOS MEA

## Abstract

The performance of electrode arrays insulated by low-temperature atomic layer deposited (ALD) titanium dioxide (TiO_2_) or hafnium dioxide (HfO_2_) for culture of electrogenic cells and for recording of extracellular action potentials is investigated. If successful, such insulation may be considered to increase the stability of future neural implants. Here, insulation of titanium nitride electrodes of microelectrode arrays (MEAs) was performed using ALD of nanometer-sized TiO_2_ or hafnium oxide at low temperatures (100–200°C). The electrode properties, impedance, and leakage current were measured and compared. Although electrode insulation using ALD oxides increased the electrode impedance, it did not prevent stable, physiological recordings of electrical activity from electrogenic cells (cardiomyocytes and neurons). The insulation quality, estimated from leakage current measurements, was less than 100 nA/cm^2^ in a range of 3 V. Cardiomyocytes were successfully cultured and recorded after 5 days on the insulated MEAs with signal shapes similar to the recordings obtained using uncoated electrodes. Light-induced electrical activity of retinal ganglion cells was recorded using a complementary metal-oxide semiconductor-based MEA insulated with HfO_2_ without driving the recording electrode into saturation. The presented results demonstrate that low-temperature ALD-deposited TiO_2_ and hafnium oxide are biocompatible and biostable and enable physiological recordings. Our results indicate that nanometer-sized ALD insulation can be used to protect electrodes for long-term biological applications.

## Introduction

The use of microelectrode arrays (MEAs) interfaced to neural structures (nerve fibers or brain areas) has gained momentum with the increasing interest to treat neurological diseases using electrical stimuli rather than pharmaceuticals. Famm (2013) introduced for this line of research and development the term “electroceuticals.” Treatment strategies using miniaturized and flexible neuroprosthetics require stable conditions, including biostable materials (Cogan, 2008; Barrese et al., 2016). The electrode lifetime may be improved by using novel materials that do not show corrosion (Wissel et al., 2018), by insulating the electrode with a thin dielectric (reviewed in Fromherz, 2002) or by a combination of both (Xie et al., 2014). Here, we characterized the dielectric insulation of TiN electrodes under the requirement to non-invasively record electrical activity. Approaches aimed to improve the electrode material were reviewed recently (Jalili et al., 2017) and are not subject of this work.

Ideally, the electrode insulation materials should be processed at low temperatures, which do not affect the electrode materials, the electrode substrate, and/or the underlying electronic circuitry. One method of choice is atomic layer deposition (ALD), which can be applied at low temperatures (100–200°C). ALD is a development of traditional chemical vapor deposition and is characterized by its cyclic pulsed character that alternates reactive and flushing steps in a half-cycle reaction. Its self-limiting and pinhole free growth allows a good deposition control, as well as ultrathin layer formation (Profijt et al., 2011; Pimenoff, 2012). High-temperature ALD oxides on silicon-based large electrodes have been used for stimulation (Schoen and Fromherz, 2007). Conversely, ALD thin film titanium nitride (TiN) electrodes have been reported recently for recording of neuronal activity (Ryynänen et al., 2019). However, the application of thin-film ALD oxides on top of recording electrodes of either passive MEAs or the translation to active, complementary metal-oxide semiconductor (CMOS)-based MEAs has not been reported.

Atomic layer deposition is a technology for depositing metals or metal oxides without defects even on complex three-dimensional structures with high aspect ratios. A further advantage of employing, e.g., titanium dioxide (TiO_2_) layers into medical implants may be the potentially enhanced biostability and biocompatibility (Yin et al., 2013). Materials with a high dielectric constant are applied in microelectronic technology as insulating layers in dynamic random-access memories and (submicrometer) field-effect transistors (Ratner and Bryant, 2004; Andriani et al., 2013).

Capacitive metal oxides have been suggested for applications in neuroscience and neural engineering for insulation of recording and stimulation electrodes (reviewed in Fromherz, 2002). Since mechanically flexible MEAs are made of polymers such as polyimide or parylene (Khodagholy et al., 2011; Ghane-Motlagh and Sawan, 2013; Stutzki et al., 2016), the deposition process of the insulators needs to be done at moderate temperatures. Plasma in ALD processes (Plasma Enhanced ALD) decreases the coating temperatures significantly, thus enabling coatings on polymers (Minton et al., 2010) or other sensitive substrates (Marichy et al., 2011). However, functional demonstration of insulated microelectrodes for neural sensing has not been reported so far. ALD-deposited films exhibit a low water vapor permeability, potentially leading to an increase in lifetime of neural implants and of *in vivo* electrophysiological systems. Insulation, on the other hand, increases the electrode impedance or conversely reduces the electrode capacitance, thus potentially limiting the recording and stimulation performance.

Here, we investigate the potential of low-temperature processed ALD to insulate TiN electrodes of passive MEAs (Stett et al., 2003) and of active, CMOS-based MEAs (Bertotti et al., 2014; Zeck et al., 2017) toward recordings of electrophysiological activity. We investigated smooth TiN electrodes, which were used as top electrodes of CMOS-based MEAs. We analyzed the recording performance and potential changes of electrical properties (impedance) after cell culture.

## Materials and Methods

### Microelectrode Arrays

Two types of MEAs were used in this work to investigate the recording performance upon insulation of electrodes using ALD-processed oxides. The first MEA type comprised compact TiN electrodes integrated into 1-mm-thick glass substrates (Stett et al., 2003). In detail, TiN (0.2 μm) for electrical traces and electrode definition was sputter deposited (Leybold Z700) and structured by reactive ion etching (RIE) (Plasmalab 800, Oxford Instruments). Silicon nitride (0.6 μm) was deposited as an insulator by plasma-enhanced chemical vapor deposition (Plasmalab 800, Oxford Instruments) at 350°C (gases: SiH_4_, NH_3_, and N_2_) and also structured by RIE. The RIE processes for TiN and for SiN were performed at room temperature (CF_4_ and O_2_ plasma). TiN was deposited by sputter coating from a TiN target with 0.2 Pa of Ar gas and 1,000 W (Leybold L560) to form a compact and smooth TiN layer. Sputtering for 30 min produced a thickness of 150 nm TiN. The electrodes were structured by lift-off with an AZ ECI 3027 resist mask. There was no additional TiN deposition to define electrodes. Electrodes of varying size (0.1024 mm^2^ to 78.5 μm^2^, corresponding to electrode sizes of 320 × 320 μm^2^, 160 × 160 μm^2^, 80 × 80 μm^2^, 40 × 40 μm^2^, 20 × 20 μm^2^; circular electrode with 10-μm diameter). The TiN electrodes themselves were later covered with ALD oxide as described below.

The second MEA type is based on a CMOS-based MEA version, which has been reported recently (Bertotti et al., 2014). However, in contrast to the reported CMOS-MEA type with capacitive electrodes (diameter: 8 μm) made of sputtered TiO_2_, here we changed the final post-processing step. On top of the compact TiN electrodes, we deposited ALD oxide, as described below.

For the creation of the capacitive recording, electrodes TiO_2_ or hafnium dioxide (HfO_2_) were deposited on the smooth TiN layer using Plasma Enhanced ALD in a direct plasma (PEALD mode, *myplas* PLASMA ELECTRONIC GmbH, Neuenbürg, Germany). The conduction traces (interconnects) of the passive MEAs were covered by Kapton foil during the ALD process. However, we cannot rule out ALD deposition on part of the SiN on the conduction trace.

Titanium dioxide was formed using a titanium tetraisopropoxide (TTIP) precursor (abcer GmbH, AB 113507) at 100°C at a thickness of 15 and 30 nm. The deposition process is based on previous work (Potts et al., 2010; Westerhausen et al., 2020).

Ti(OCH3)7+436O*→TiO+212CO+214HO2

Titanium tetraisopropoxide was introduced into the chamber using three pulses of 30-ms duration each (80-ms pause between the pulses). Argon was flushed at 2 sccm during and after the TTIP pulses for 800 ms.

The plasma was generated with 22 sccm oxygen for a duration of 1 s and with a power of 135 W. Deposition occurred at 100°C with a layer growth of 0.04 nm per cycle. After the contacts for EIS measurements were covered using Kapton tape (Intertape 4118), ALD coating was started. Layer thickness was verified using a reflectometry system (NanoCalc, Ocean Optics Inc., Largo, FL, United States). For an oxide thickness of 30 nm, we used 726 cycles (total coating duration: 70-min duration), whereas for the thickness of 15 nm, we used 363 cycles (35 min).

### ALD of HfO_2_

Hafnium dioxide was deposited from hafnium(iv)-t-butoxide (HTB) (abcer GmbH, AB 106072) at 200°C at a thickness of 15 nm with a growth 0.07 nm per cycle, using the same setup and plasma configuration as described above.

CH16H36fO+448O*→HfO+216CO+218HO2

Hafnium(iv)-t-butoxide (suggested by Lao et al., 2005) was introduced into the chamber using three pulses of 30-ms duration each (250-ms pause between the pulses). Argon was flushed at 10 sccm during and after the TTIP pulses for 1,000 ms. The plasma was generated with 19 sccm oxygen for a duration of 2.5 s and a power of 115 W. The total deposition (22 min) comprised 200 cycles.

### Cell Culture of Cardiomyocytes, of Neuronal Cultures, and of *ex vivo* Retina

The experimental procedures for the preparation of cardiomyocytes, of neuronal cell cultures, and of the *ex vivo* mouse retina were performed in compliance with the institutional guidelines of the Natural and Medical Sciences Institute and were reported to the local authorities (Regierungspräsidium Tübingen, Germany).

Prior to seeding of cardiomyocytes, of neuronal cultures, or of interfacing *ex vivo* retina, both MEA types were pretreated for 30–60 s in a Plasma Cleaner (∼10 W, PDC-32G, Harrick Plasma) to obtain a hydrophilic surface.

The preparation of cardiomyocytes was based on an earlier report (Connolly et al., 1990; Gerwig et al., 2012). Cardiac myocytes were prepared by enzymatic dissociation from 13-day chick embryos. Hearts were removed, ventricles minced, and dissociated in four to five cycles of trypsin at 37°C. After discarding the remaining coarse component, the cells are prepared and diluted in F-12 medium (Gibco 21765-029) with 20% fetal bovine serum (FBS) (SIGMA F7524) at a density of 20,000 cells/μL. Cultivation of cardiomyocytes is done in Dulbecco modified eagle medium (DMEM) (GE Hyclone SH30285.01) with 3% FBS. Finally, they were plated on MEAs, previously sterilized with 70% ethanol overnight, and coated with 6 μL of nitrocellulose (5% diluted in methanol). The cell concentration ensured a good coverage of all electrodes by a single layer of cells. Cell cultures were incubated at 37°C and 5% CO_2_. The medium (DMEM with 3% FBS) was changed every 2–3 days. Extracellular recordings were performed after 5 days of *in vitro* culture.

Primary neuronal cultures were prepared based on a recent report (Kriebel et al., 2020). Briefly, cortical neurons were prepared from E18 rat embryos of either sex by trypsin digestion and subsequent trituration of respective tissues. Cells were seeded in serum-free MEM with B27 supplement (Thermo Fisher Scientific, Waltham, MA, United States) on polyethyleneimine-coated MEAs at a density of 1.5 × 10^5^ cells/cm^2^. At 1 day *in vitro* (DIV1), the plating medium was replaced by fresh serum-free MEM with B27 supplement. Cultures were maintained at 37°C, 5% CO_2_ in serum-free MEM with B27 supplement until DIV3 followed by a 50% medium change with BrainPhys Neuronal Medium containing SM1 supplement (STEMCELL Technologies). A 50% medium change using BrainPhys Neuronal Medium containing SM1 supplement was performed every other day. Fluorescent labeling of cultured neurons was achieved by viral transduction at DIV3 (MOI 1 × 10^4^ VG/cell) employing AAV suspensions raised by the Viral Core Facility of the Charité – Universitätsmedizin Berlin using pAAV_CamKIIa_EGFP_miR338_3p (kindly provided by S. Zakharenko, St. Jude Children’s Research Hospital). Impedance measurement and fluorescence imaging (upright microscope, Axio Imager, Zeiss) were performed between DIV 14 and 16.

The preparation of *ex vivo* C57Bl6/J retina was performed based on previous reports (Menzler et al., 2014; Stutzki et al., 2016). Briefly, eyes were enucleated, and retinas were isolated and dissected in Ames’ medium (A 1420, Sigma-Aldrich) under dim red light. A retinal portion was flat-mounted in epiretinal configuration to the coated surface of the CMOS MEA chip. After each use, the CMOS MEAs are cleaned with Terg-a-zyme (Sigma-Aldrich, Z273287) dissolved in bidistilled water. Prior to the interfacing of the retina, they are pretreated using air plasma, as described above, and coated with poly-L-lysine (Sigma-Aldrich, P2636, 1 mg/mL dissolved in bidistilled water) for at least 30 min. The recordings with CMOS MEA chips insulated with ALD-processed HfO_2_ were repeated more than 10 times without signs of deterioration.

### Impedance Measurement

For electrical impedance and phase characterization, a three-electrode setup was used utilizing an external platinum counter electrode (area of 2.5 cm^2^) and an Ag/AgCl-reference electrode (WPI, Sarasota, FL, United States). All impedance measurements were performed in electrolyte over a frequency range of 100 mHz to 1 MHz, whereas phase behavior was analyzed over a range of 100 Hz to 1 MHz using a Parstat 2263 (AMETEK Princeton Applied Research, Oak Ridge, TN, United States). The passive MEA had been pre-cleaned using an O_2_-plasma. The MEA was placed in a customized holder to enable top side contact of individual electrodes after a Makrolon chamber was glued (elastosil 41) to the substrate and filled with 2 mL of phosphate-buffered saline (PBS). A mean impedance value from six electrodes was calculated for electrodes of 320 × 320 and 160 × 160 μm^2^, respectively. To compare the change in impedance after primary neuronal cell culture, we calculated the ratio of impedances as follows: (*Z*_2_−*Z*_1_)/(*Z*_2_+*Z*_1_). Here, *Z*_2_ represents the impedance at 1 kHz after cell culture, whereas *Z*_1_ represents the value of the same electrode prior to cell culture.

### Capacitance Measurement

The electrodes capacitance was measured using the three-electrode setup at the Parstat 2263 after the substrate had been pre-cleaned using an O_2_-plasma. A DC voltage of −0.5 to +3 V at 1 kHz was applied in 50-mV steps with a duration of 5 s for each value. Stray capacitance resulting from the conducting pathways and measurement setup was identified by extrapolation and subtracted from the detected specific capacitance values. Frequency-dependent capacitance measurements were performed at frequencies of 10 kHz, 1 kHz, 200 Hz, and 25 Hz. The mean capacitance averaged over all electrodes of the same area (*n* = 6 for each area) is reported.

### Leakage Current

Leakage current was measured using the Keithley 236 (Keithley Instruments GmbH, Solon, OH, United States) after the substrate had been pre-cleaned using an O_2_-plasma. The MEA was contacted using a customized holder with the internal reference electrode of 7.81 mm^2^ serving as a reference point. During the measurement in PBS, a DC voltage of +5 to −3 V was applied in −0.5-V steps of 5-s duration each. Leakage current was measured for large electrodes (320 × 320 μm^2^).

### Electrophysiological Recording

Recording of electrophysiological activity was performed using the MEA types described above. Recordings of spontaneous electrical activity from cultured cardiomyocytes were performed using a passive glass MEA. Signals were amplified using a commercial MEA amplifier (MEA 2100, Multi Channel Systems MCS GmbH, Reutlingen, Germany) at 37°C. The temperature was adjusted using a controlled heating plate underneath the MEA (Temperature Controller 02, Multi Channel Systems MCS GmbH). Extracellular signals were further analyzed using MC_Rack software. Second, light-induced electrical activity of retinal ganglion cells was recorded using a CMOS-based MEA (CMOS MEA 5000, Multi Channel Systems MCS GmbH, Reutlingen, Germany). *Ex vivo* mouse retina (strain: C57/Bl6J, Charles River, Germany) was interfaced to the CMOS MEA as described in Menzler et al. (2014) and Bertotti et al. (2017). The retina was stimulated using an LED with a peak sensitivity at 470 nm (μ-matrix form Rapp Optoelectronics GmbH; pE-4000 from CoolLED). The light intensity was 100 μW/mm^2^ (estimated with Ava AvaSpec-ULS2048, Mountain Photonics GmbH, Landsberg am Lech, Germany).

## Results

### Impedance and Phase of Electrodes Coated With Low-Temperature Dielectric

**FIGURE 1:**
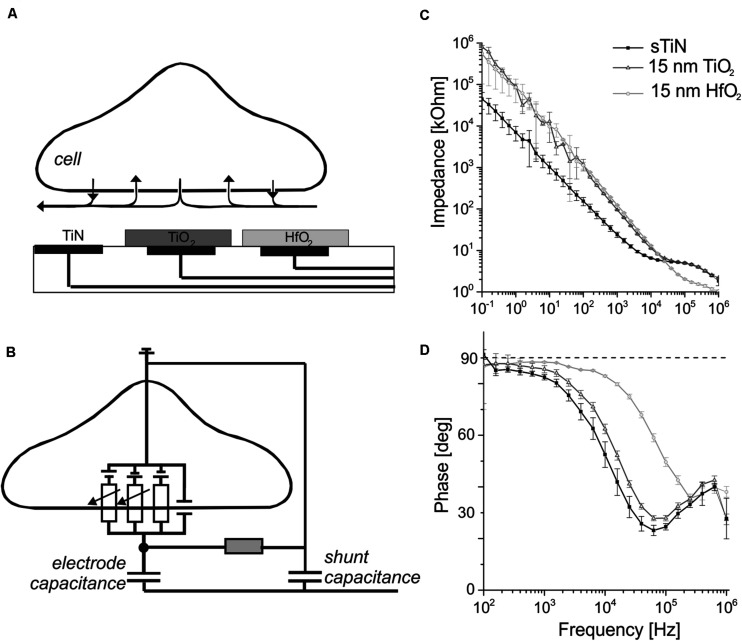
Schematic of the capacitive electrodes used for neural sensing with their impedance and phase behavior. **(A)** A simplified schematic cross section of the utilized capacitive microelectrode array in contact with an electrogenic cell. Electrodes insulated with ALD-TiO_2_ or ALD-HfO_2_ are compared here with bare, compact TiN electrodes. **(B)** Schematic cross section showing a simplified equivalent circuit of an interfaced electrogenic cell, a capacitive sensing electrode, seal resistance, and shunt capacitance. Figure modified from rf. (Zeck et al., 2017). **(C)** Impedance measurement of the capacitive electrode with 15 nm TiO_2_ (dark gray symbols) and 15 nm HfO_2_ (light gray) and for the uncovered smooth TiN base electrode for comparison (black trace). **(D)** Phase angle of TiN stimulation electrode covered with 15 nm of HfO_2_ (light gray) or TiO_2_ (dark gray). Black symbols denote the phase shift for bare smooth TiN.

The amplitude of the recorded extracellular voltage caused by active electrogenic cells depends, among others, on the capacitance/impedance of the transducing electrode. The recording principle of extracellular activity is depicted in Figure 1A. Electrical activity of electrogenic cells changes the extracellular voltage above an electrode due to the ionic current flow through membrane-bound ion channels. The voltage above the electrode is transduced by the electrode capacitance (Figure 1B). Ideally, this signal is amplified and detected. However, the shunt capacitance of the insulated conduction leads (interconnects) may attenuate and change the detected signal. The impedance of ALD-coated electrodes were measured and calculated as described in section “Materials and Methods”. Here, mostly large electrode areas were investigated, to avoid the effect of shunt capacitances, estimated to be about 150 pF (Jones et al., 2020). Our estimate is based on the area of the electrical trace (1 mm^2^), thickness of the silicon nitride insulator (600 nm), and its electric permittivity of 7.

The frequency-dependent impedance for electrodes of 1.024 × 10^–3^ cm^2^ area (square-shaped electrode with 320-μm side length) at frequencies between 100 mHz and 1 MHz is shown in Figure 1C. Electrodes covered with 15-nm ALD-TiO_2_ and 15-nm ALD-HfO_2_ were compared to compact, smooth TiN. At 1 kHz, the impedance for smooth TiN electrodes is 24.1 ± 3.46 kΩ (mean ± standard deviation). Upon insulation with TiO_2_ (15 nm), the impedance increases almost fourfold to 95.5 ± 1.63 kΩ, while upon insulation with HfO_2_ (15 nm), it increases fivefold to 123.35 ± 2.21 kΩ.

The capacitive property of the electrodes is best visualized using the impedance phase plots (Figure 1D). At 1 kHz, which represents the dominant frequency of single-cell electrical activity, all three electrode types showed a nearly capacitive behavior. The detected phase angle for smooth TiN is 82.5 ± 1.1 degrees, whereas insulation with 15 nm of TiO_2_ increased the value to 85.6 ± 1.4 degrees. A similarly high value was obtained after insulation with HfO_2_ (15 nm): 88.3 ± 0.3 degrees. For high frequencies (10 kHz), the phase angle decreases for smooth TiN and for TiO_2_, but remains at a value of 82.94 ± 0.3 degrees for HfO_2_.

In summary, the presented electrodes show capacitive behavior up to frequencies of ∼1 kHz for TiN and TiO_2_ and up to 10 kHz for HfO_2_. In the following, we measure the specific capacitance of the three electrodes.

### Specific Capacitance of TiN Electrodes Coated With ALD Dielectric

**FIGURE 2:**
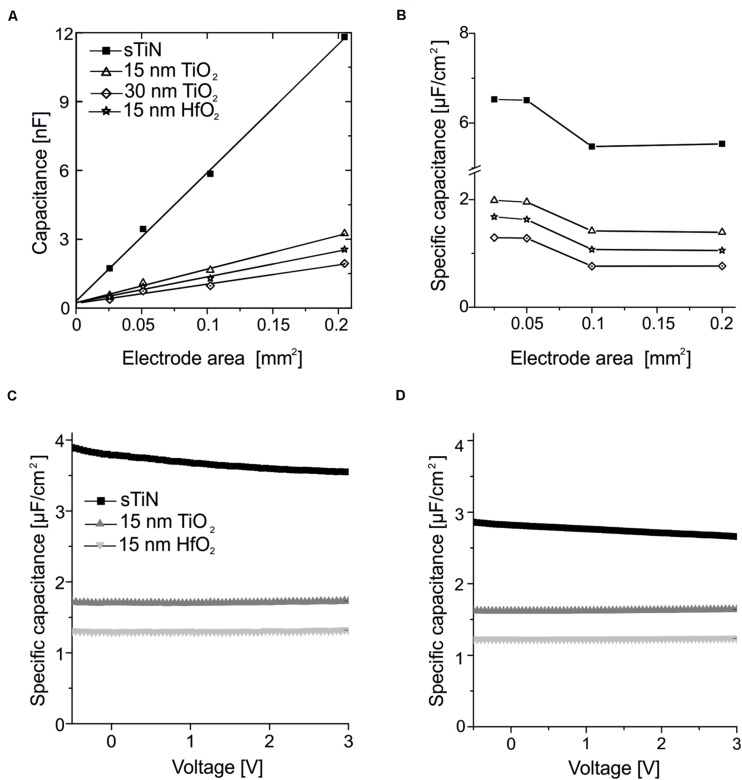
Characterization of the capacitance of the ALD-insulated recording electrodes. **(A)** Linear dependence of capacitance with electrode area measured using smooth TiN electrodes insulated with 15 or 30 nm of ALD-TiO_2_, or electrodes insulated with 15 nm ALD-HfO_2_. The data are compared to uncoated smooth TiN electrodes. Measurements were performed at a frequency of 1 kHz. The linear fit has a small offset (0.2 nF). **(B)** Specific capacity over area for smooth TiN with 15 or 30 nm of ALD-TiO_2_, as well as 15 nm ALD-HfO_2_ compared to the uncoated smooth TiN electrode at a frequency of 1 kHz. **(C)** Stable behavior of specific capacitance irrespective of the applied DC voltage applied to the electrode. Measurements were performed at 25-Hz stimulation frequency for 15 nm HfO_2_ (light gray), TiO_2_ (dark gray) on smooth TiN compared to uncoated TiN electrodes (black line) on large electrodes (320 × 320 μm^2^). **(D)** Same analysis as in panel **(C)** except that here a test frequency of 1 kHz was used.

The absolute and area-specific values of the electrode capacitance are presented in Figures 2A,B. The absolute capacitance increases linearly with electrode area for all tested ALD oxides. We note that there is a minor offset of 0.2 nF, which we attribute to parasitic capacitances of our measurement setup. This also prevents estimation of the capacitance parameters for small electrodes.

Results for electrodes with 15 or 30 nm of ALD-TiO_2_ as well as 15 nm HfO_2_ are compared to the uncoated TiN electrode. The specific capacitance evaluated for the 15-nm layer of ALD-TiO_2_ is 1.64 μF/cm^2^ (estimated from a 0.1-mm^2^ electrode). This value is slightly higher than that from a literature report for ALD-TiO_2_ (1.48 μF/cm^2^) deposited at 300°C on conductive silicon (Wallrapp and Fromherz, 2006). At 30 nm TiO_2_, both the calculation from impedance measurement and the voltage-dependent direct measurement provide a specific capacitance of 0.99 μF/cm^2^. This value is higher than the theoretically expected value of 0.82 μF/cm^2^ if one would assume a linear dependence with oxide thickness. This result suggests that a thin oxide layer may exist between the electrode surface and ALD insulation. The cited literature value refers to remote plasma ALD layers, whereas here with direct plasma ALD is deposited, which results in layers that are more compact and thus a slightly increased capacity. For TiO_2_, an increasing dependence on capacitance and frequency can be observed when high frequencies are used at high specific capacitances, due to the symmetrical error of the series resistors of the measurement setup (Aarik et al., 2012).

The specific capacitance evaluated for the 15-nm layer of ALD-HfO_2_ ranges between 1.1 and 1.6 μF/cm^2^ for the different electrode areas. The range of estimated values (electrode areas: 0.025–0.2 mm^2^) may be explained by the inaccuracies of electrode sizes used for capacitance estimation. The specific capacitance shows no voltage dependence, i.e., remains stable irrespective of the applied DC electrode voltage (Figure 2C; range = −0.5 to 3 V). The specific capacitance shows little frequency dependence, with a decrease of less than 10% from 25-Hz probing stimulus to a 1-kHz probing stimulus (Figures 2C,D). Further values of the specific capacitance, evaluated at 200 Hz and at 10 kHz, are provided in the Table 1. The shunt capacitance resulting from the insulator covering the conducting pathways and the measurement setup were previously estimated by extrapolation and subtracted.

**TABLE 1:** Specific capacitance at different frequencies (25 Hz, 200 Hz, 10 kHz, and 10 kHz) for 15 nm HfO_2_ or TiO_2_ on smooth TiN electrode (320 × 320 μm^2^) compared to the specific capacity of uncoated electrodes of both fractal and smooth TiN.

**Electrode**	**Frequency [Hz]**	**Specific capacitance [μF/cm^2^] at 0 V**
TiN (fractal)	25	110.96
	200	92.77
	1,000	75.8
	10,000	57.01
TiN (smooth)	25	3.79
	200	3.57
	1,000	2.82
	10,000	3.6
15 nm TiO_2_	25	1.17
	200	1.66
	1,000	1.62
	10,000	1.62
15 nm HfO_2_	25	1.29
	200	1.26
	1,000	1.22
	10,000	1.15

### Leakage Current

**FIGURE 3:**
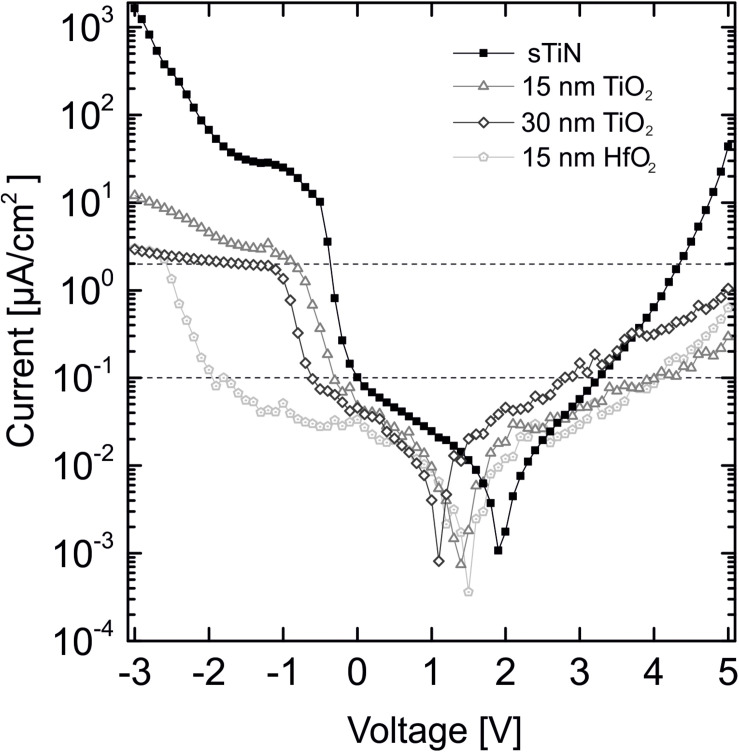
Leakage current behavior of 15 and 30 nm TiO_2_ on smooth TiN compared to 15 nm HfO_2_ on TiN and an uncovered TiN electrode at an applied voltage range of +5 V to –3 V. Displayed threshold values of 0.1 μA/cm^2^ at ±0.8 V [dynamic random access memory (DRAM) applications, International Technology Roadmap for Semiconductors (Aarik et al., 2012)] and the 2 μA/cm^2^ lowest stimulation threshold limit for cell stimulation (Eickenscheidt et al., 2012).

The leakage current is a second important characteristic of an insulated electrode, as it determines electrode stability. The leakage current density through the dielectric ALD layer of the electrode is shown in Figure 3. A DC voltage (range = +5 V to −3 V) is applied to individual electrodes. Leakage currents are measured for electrodes coated with 15 or 30 nm of TiO_2_ on smooth TiN, for electrodes coated with 15 nm HfO_2_ layer, and for electrodes with uncoated smooth TiN electrodes. Both ALD coatings reduce the leakage current over a relatively wide range. The strong increase of the leakage current at bias voltages below −1.8 V might be caused by electrochemical reactions (gas bubble formation) as reported previously (Patan et al., 2006).

For the possible application of the characterized stimulation electrodes in neuronal implants, specific threshold values for leakage current density need to be considered. For DRAM applications, a leakage current density of less than 0.1 μA/cm^2^ [International Technology Roadmap for Semiconductors (ITRS)] in the voltage range ±0.8 V is required (Aarik et al., 2012). Our leakage currents fulfill this requirement. The leakage current minima are shifted because of small offset voltages during measurement, which are not corrected for. If electrodes would be used for electrical stimulation, the leakage current must be below the stimulation threshold limit for activation of electrogenic cells [<2 μA/cm^2^, (Eickenscheidt et al., 2012)]. The oxides fulfill this requirement up to 4 V as shown by the upper dotted line in Figure 3. The horizontal shift of the minimum values is caused by uncontrolled small potentials at the electrode contact pads.

We summarize that both low-temperature oxides show leakage currents below the required values. Insulation with hafnium oxide is superior to TiO_2_ with respect to the leakage current.

### Electrophysiological Signals From Cardiomyocytes Recorded by ALD-Coated Electrodes

**FIGURE 4:**
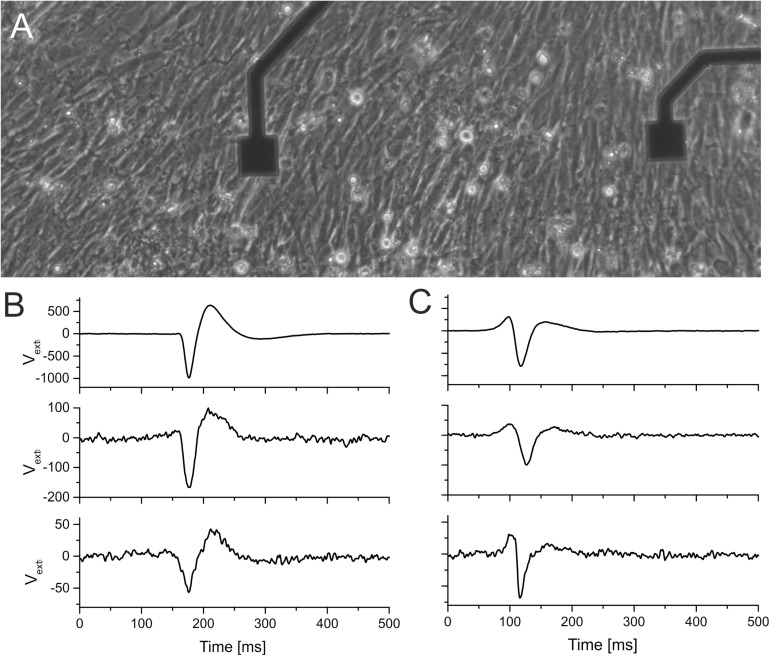
Comparison of signal shapes recorded by TiN electrodes and by insulated (ALD-coated) TiN electrodes. **(A)** Micrograph of cardiomyocytes grown on HfO_2_-coated smooth TiN microelectrodes. The electrodes have an edge size of 40 μm. **(B,C)** Extracellular signals recorded on three different electrodes. The first row in panels **(B,C)** shows signals recorded using a commercial electrode (fractal TiN). The second row in panels **(B,C)** shows signals recorded on electrodes where the smooth TiN had been coated with 15 nm of TiO_2_. The third row shows signals recorded on smooth TiN electrodes coated with HfO_2_.

To test the application of electrodes insulated with ALD oxide in electrophysiology, we cultured cardiomyocytes for 5 days (details described in section “Materials and Methods”). Stable and homogenous cultures were obtained on all oxides, as shown in Figures 4A–C. To investigate the biocompatibility of ALD coating, we recorded electrophysiological activity from these cultures. On all MEAs coated with ALD oxides (TiO_2_ and hafnium oxide, respectively), we recorded electrophysiological activity. We compared the signal shapes and the beating frequency of the cardiomyocyte culture. The signal shapes were qualitatively similar on MEAs coated with oxide as compared to recordings from conventional MEAs for both TiO_2_ (Figure 4B) and HfO_2_ (Figure 4C). The smaller signal amplitude is attributed to the signal attenuation by the shunt capacitance across the electrode connecting lead (Figure 1B). The voltage divider between recording electrode impedance (1.5 MOhm at 1 kHz, corresponding to 100 pF) and the shunt capacitance [∼150 pF, (Jones et al., 2020)] reduces the signal amplitude significantly. The beating frequency of the cardiomyocytes recorded on ALD oxide-coated MEAs was the same as the beating frequency recorded on conventional TiN MEAs (data not shown).

### Light-Induced Electrophysiological Signals From *ex vivo* Retina on CMOS-Based MEAs

**FIGURE 5:**
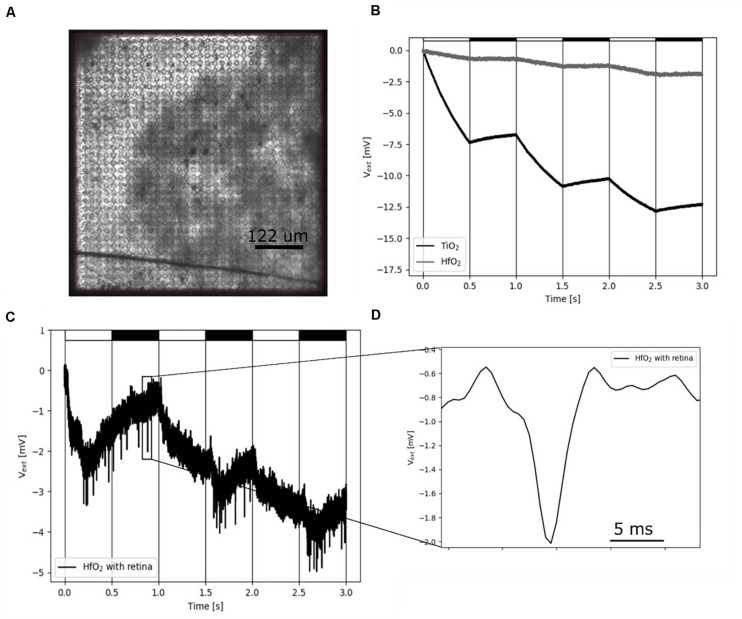
Recording of electrical single-cell activity using hafnium oxide-coated CMOS MEA. **(A)** Photograph of the CMOS sensor array with interfaced retina. **(B)** Light-induced drift by illumination using a 460-nm LED on sputtered TiO_2_ and low-temperature HfO_2_ ALD-coated CMOS-MEAs. The LED is pulsed with a cycle of 1-Hz, on-off periods represented by white and black rectangles. **(C)** Low-pass filtered (1.5 kHz) HfO_2_ ALD-coated CMOS MEA sensor signal. The sensor signal shows spikes of a retinal ganglion cells upon stimulation with 460-nm 1-Hz flicker. **(D)** Zoom of one single extracellular retinal action potential from panel **(C)**.

To demonstrate the benefit of ALD-coated electrodes, we investigated the insulation of CMOS-based MEAs (Bertotti et al., 2014; Thewes et al., 2016 in experiments with *ex vivo* flat-mount retinal tissues (Figure 5A). If these arrays are coated with sputtered TiO_2_, they exhibit a strong light dependence, which corresponds to the energy band gap of 3.0 eV (Bertotti et al., 2017). Light-induced physiological retinal activity can be recorded under these conditions for short time periods only (Figure 5B), before driving the sensor in saturation. Here, we therefore coated the CMOS MEAs with hafnium oxide instead of TiO_2_. Hafnium oxide has an energy band gap of 5.25–5.8 eV (Shaimeev et al., 2007) and is therefore not sensitive to visible light. We first demonstrated the ability of such CMOS MEAs to record light-induced electrophysiological activity from retinal ganglion cells in the explanted *ex vivo* flat-mounted mouse retina (Figure 5C). Repetitive light pulses (1 Hz) evoke action potentials in retinal ganglion cells, which are detected by the CMO sensors as negative voltage deflections (spikes). Single spikes have the same waveform shape as spikes recorded with TiN electrodes (Figures 4B,C), although with CMOS MEAs, usually larger amplitudes are detected as compared to passive MEAs (Zeck et al., 2017). The CMOS MEA recording electrode (diameter: 8 μm) has a capacitance of about 1 pF (assuming a specific capacitance of 2 μF/cm^2^). The shunt capacitance for the CMOS MEA has been estimated to be as low as 0.13 pF (Bertotti et al., 2016); therefore, little attenuation is expected. More importantly, hafnium oxide-coated CMOS MEAs exhibit a reduced light sensitivity, which makes them attractive for future applications, including the combination of electrical recording upon strong optogenetic stimulation (Ferrari et al., 2018; Reh et al., 2018).

### Long-Term Biocompatibility of TiN Electrodes Insulated With ALD Oxides

**FIGURE 6:**
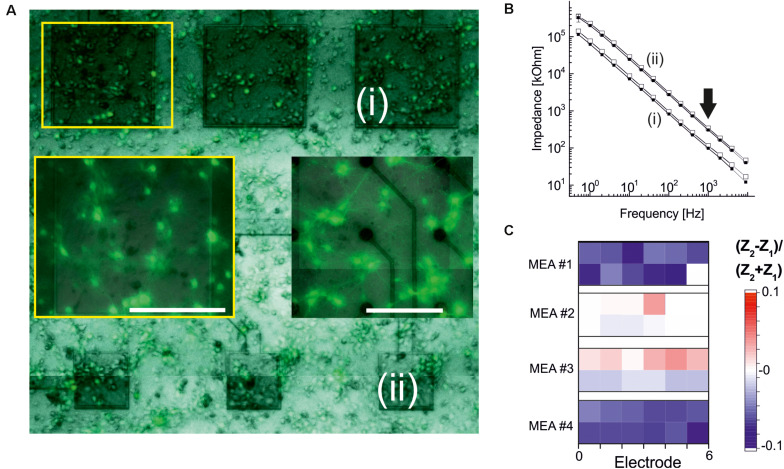
Electrode stability after primary neuronal cultures. **(A)** Microscopic fluorescence image of an ALD-HfO_2_-insulated MEA after 2 weeks in culture. Three large electrodes [(i), 320 × 320 μm^2^; dark gray] and three smaller electrodes [(ii), 160 × 160 μm^2^] are covered by primary neuronal networks. Left insert marked by yellow rectangle: Fluorescence image at higher magnification showing homogenous networks. Right insert: Fluorescence image of a control culture on a passive MEA with fractal TiN electrodes (Stett et al., 2003). Scale bars: 200 μm. **(B)** Impedance spectra recorded for the electrodes marked in panel **(A)**. Smaller impedances are recorded for the large electrodes (i). Open symbols denote impedance values before cell culture; filled symbols denote impedance values after cell culture. Arrow indicates toward impedances at 1 kHz. **(C)** Quantitative evaluation of relative changes in impedance at 1 kHz after cell culture. Blue symbols denote a decrease in impedance; red, an increase. Four MEAs were evaluated, and for each MEA, six large electrodes (320 × 320 μm^2^; lower row) and six smaller electrodes (160 × 160 μm^2^) were considered.

To assess the long-term biocompatibility and biostability of electrodes coated with ALD-TiO_2_ or with ALD-HfO_2_ further, we cultured primary neurons for 2–3 weeks (see section “Materials and Methods”). The appearance of neuronal cultures on passive MEAs insulated with ALD- HfO_2_ was qualitatively indistinguishable from the appearance on control MEAs with commercial, passive MEAs comprising fractal TiN electrodes (Figure 6A).

To obtain a qualitative assessment of the stability of the ALD oxide-coated electrodes, we measured the impedance of selected electrodes (320 × 320 μm^2^ and 160 × 160 μm^2^) prior to neuronal cell culture and after neuronal cell culture. Electrodes were coated either with 15 nm ALD-TiO_2_ or with 15 nm ALD-HfO_2_. Example traces of the impedance of two ALD-HfO_2_ insulated electrode sizes are shown in Figure 6B. We next compared the impedances at 1 kHz, which represents the optimal frequency for recording of neuronal activity. There was a small but significant (*p* < 0.05, *t* test) decrease in impedance by 3.7%, calculated for 48 electrodes (Figure 6C) after 14–16 days in culture. In summary, the electrophysiological experiments demonstrate the biocompatibility and biostability of the low-temperature ALD oxides, as well as the benefit for the recording of light-induced neural activity. Future investigations, including *in vivo* long-term evaluations, are necessary to obtain a conclusive answer about their long-term stability.

## Discussion

Here, we investigated the use of low-temperature ALD insulation of TiN electrodes in physiological recordings. Although the coating reduced the electrode capacitance and thus increased its impedance, extracellular action potentials could be reliably recorded on passive MEAs. For CMOS-based MEAs, the small electrode capacitance does not cause signal attenuation because of the even smaller shunt capacitance in the active electrode pixel. We further provided a use case, where ALD oxide protected the light-sensitive CMOS structure and thus enabled improved recording of light-sensitive neural tissue.

Titanium nitride is a conductive electrode material with many advantages. It has a high chemical and mechanical stability, good conductivity, and biocompatibility, as well as a large potential window (Weiland et al., 2002; Cogan, 2008). For *in vitro* electrophysiology, this material provides good results, and MEAs with TiN electrodes can be reused several times. *In vivo* TiN electrodes have been used as stimulation electrodes of the subretinal implant Alpha IMS with time to failure of 14.6 months (Daschner et al., 2018). We suggest that for *in vivo* application, for long-term cell cultures application or for combined optical stimulation and electrical recordings (Kozai and Vazquez, 2015), additional protective layers made of ALD oxides may be used.

We discuss several approaches to improve capacitive electrodes. The large influence of parasitic capacitances on small electrodes represents an obstacle for passive MEAs (Figures 4B,C) but apparently not for CMOS-based MEAs (Figure 5). A possible solution would be to effectively increase the surface area of the electrodes by changing the specific topography without increasing their actual space requirement on the MEA. Approaches to produce three-dimensional structured electrodes and substrates already exist (Heuschkel et al., 2002), but have not been combined with ALD insulation. Optimization of the ALD oxide itself could contribute to an improvement of the capacitive electrode system. For example, the choice of another ALD precursor for TiO_2_ deposition could positively influence the leakage current behavior of the oxide layer. Here, we used TTIP in combination with O_2_ plasma, but higher permittivity for layers of TiCl_4_ precursor with H_2_O as the second reaction gas (Aarik et al., 2012) has been reported. Adequate doping of TiO_2_ with aluminum could decrease the leakage current, although the permittivity of the oxide is reduced (Aarik et al., 2012). An O_2_ plasma pretreatment of the base electrode could positively influence the layer growth by reducing impurities and improving the stoichiometric oxygen ratio at the electrode surface, which would result in a reduction in leakage currents. A final annealing of the oxide layer at 300°C could also reduce leakage current (Fröhlich et al., 2011; Hudec et al., 2011) but may interfere with the goal of low-temperature processing. A reduced shunt capacitance (impedance) of the conducting traces seems indispensable for passive MEAs. This may be achieved by shorter conducting traces and by smaller culture chambers.

Although electrode insulation may enable long-term recording, the reduction of electrode capacitance reduces their use for electrical stimulation. The specific capacitance (∼1–2 μF/cm^2^) of ALD oxides is too low to be used for efficient electrical microstimulation, which is characterized by stimulus pulses between 0.1 and 5 ms and stimulation charge densities ranging between 0.01 and 1 C/cm^2^ (Cogan et al., 2016; Corna et al., 2018). Electrodes with fractal surface topography and thus higher active area may be coated with ALD oxides and tested in the future.

ALD oxide coating of microelectrodes may, however, be very beneficial for *in vitro* recording extending over several months (Odawara et al., 2016) or may be applied to light-sensitive neural recordings, which need to deal with inherent light sensitivity of the recording electrode (El Hady et al., 2013). Specifically, in CMOS-based application, the insulation with CMOS-compatible and light-protecting ALD oxides opens new experimental opportunities. CMOS-based MEAs have lower shunt capacitances compared to the electrode capacitance, and therefore no signal attenuation occurs (Frey et al., 2010; Bertotti et al., 2016). The combination of electrical recording and photostimulation will enable cell type-specific stimulation of optogenetically transduced neurons and simultaneous recording of network activity (Rivnay et al., 2017; Ferrari et al., 2018).

## Data Availability Statement

All datasets presented in this study are included in the article/supplementary material.

## Author Contributions

VB and GZ designed the study. MD and MR performed the experiments and analyses. MM assisted with ALD oxides. GH was instrumental in processing of the TiN electrodes and MK was in charge for the neuronal culture and Figure 6A. GZ drafted the manuscript with input from all authors. All authors reviewed the final manuscript.

## Conflict of Interest

The authors declare that the research was conducted in the absence of any commercial or financial relationships that could be construed as a potential conflict of interest.
